# Takotsubo Cardiomyopathy Triggered by MRI-Induced Fear and Anxiety

**DOI:** 10.1155/cric/2612009

**Published:** 2025-04-27

**Authors:** Ahmed Hassaan Qavi, Andreea Constanta Stan, Ateet Kosaraju, Rony L. Shammas

**Affiliations:** ^1^Department of Interventional Cardiology, East Carolina University Health Medical Center, Greenville, North Carolina, USA; ^2^Department of Medicine, Sanford USD Medical Center, Sioux Falls, South Dakota, USA; ^3^Department of Cardiology, FirstHealth of the Carolinas, Pinehurst, North Carolina, USA

**Keywords:** echocardiography, MRI, stress cardiomyopathy, Takotsubo cardiomyopathy

## Abstract

Takotsubo cardiomyopathy (TC), or ‘broken heart syndrome,' is marked by transient left ventricular dysfunction in the absence of acute, severe coronary artery disease that explains the pattern and degree of LV dysfunction. Both emotional and physical stressors have been associated with TC. We present a case of TC that was precipitated by a routine magnetic imaging resonance scan, highlighting a rare and previously unreported trigger for TC.

## 1. Introduction

Takotsubo cardiomyopathy (TC), also known as “stress cardiomyopathy,” “broken heart syndrome,” and “transient apical ballooning syndrome,” is characterized by acute, usually transient left ventricular dysfunction, typically involving apical and mid portions of the left ventricle. While classically described in the absence of obstructive coronary artery disease (CAD), TC can coexist with CAD in 1 out of 4 cases [1]. Postmenopausal women account for up to 80%–90% of all cases [1, 2]. Although the exact mechanism remains poorly defined, TC has been attributed to transient hyperadrenergic state associated with emotional or physical stressors. [1–6] Herein, we present a unique case of TC precipitated by a routine magnetic imaging resonance (MRI) scan of the brain, shedding light on this rare but potentially serious complication of MRI imaging.

## 2. Case Description

A 76-year-old white female presented with syncope after one week of nausea, emesis, and poor appetite. She has a history of CAD status post remote coronary artery bypass surgery (left internal mammary artery (LIMA) graft to left anterior descending artery (LAD), and saphenous venous grafts (SVG) to right coronary artery (RCA) and first obtuse marginal artery (OM1)), benign positional vertigo, chronic intermittent hyponatremia, untreated anxiety, Type 2 diabetes, and hypertension. Her home medications included aspirin, lisinopril–hydrochlorothiazide, and metoprolol tartrate.

**Figure 1:**
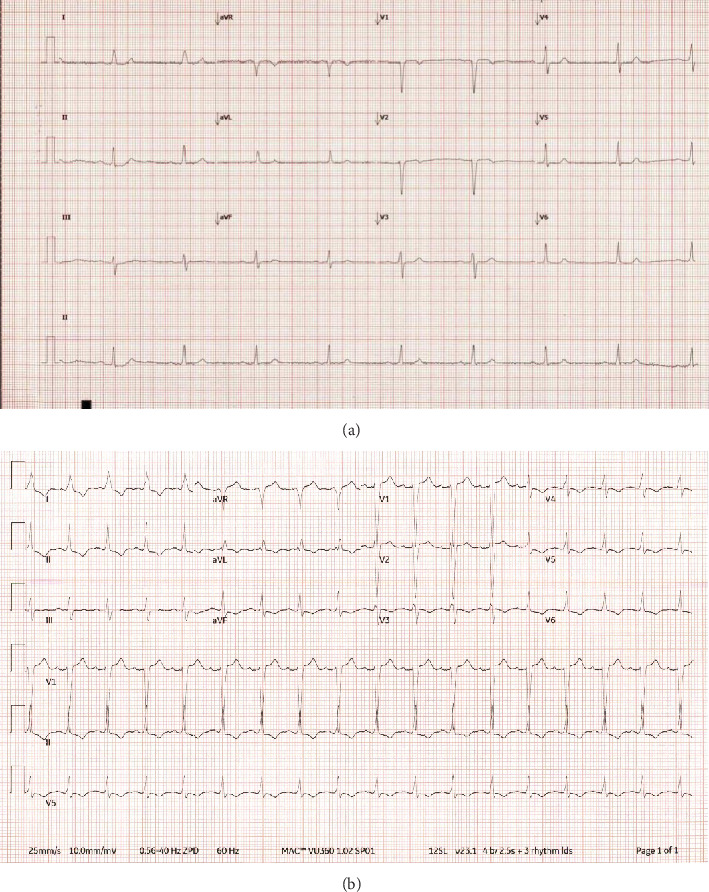
(a) Baseline electrocardiogram showed sinus bradycardia at 53 beats per minute. (b) Electrocardiogram at the time of event showed worsening ST depression and T-wave inversion in the inferior and anterolateral leads.

On presentation, her blood work was pertinent for severe hyponatremia (Na = 119 mmol/L, reference 135–145 mmol/L), decreased magnesium at 1.2 mg/dL (reference 1.7–2.2 mg/dL), and nondiagnostic high sensitivity troponin-I (hsTnI) at 14 and 17 ng/L (reference 12 ng/L or less). Admission electrocardiogram (ECG) showed sinus bradycardia at 53 beats per minute (Figure 1a) and chest X-ray was unremarkable.

Two days after admission, her Na increased to 130 mmol/L and she underwent a transthoracic echocardiogram (TTE) which showed normal left ventricular ejection fraction (LVEF) and no wall motion abnormalities (WMA) (Video S1). The evening after her echocardiogram, she proceeded to have a brain MRI scan. Shortly after arrival back to the floor from her MRI, she became acutely short of breath and hypoxic. Stat chest X-ray showed new onset pulmonary edema. ECG showed incomplete left bundle branch block with mild ST elevation in leads V1 and V2 and repeat ECG few hours later showed worsening ST depression and T-wave inversion in the inferior and anterolateral leads (Figure 1b). Cardiology consultation was requested next day, and a limited echocardiogram revealed new finding of severe LV dysfunction with severe hypokinesis to akinesis of the apex and mid-distal anterior, septal, lateral segments with sparing of basal walls (Video S2). No echocardiographic evidence of left ventricular outflow obstruction was noted. Upon specific questioning, the patient stated that this was her first MRI ever and that she was quite apprehensive and fearful during the scan, especially conveying her heightened unease and discomfort caused by the machine's noise. However, she refrained from notifying anyone, fearing potential disruption to the study. hsTnI checked after this event increased: 548 > 1691 > 2136 ng/L. B-type natriuretic peptide (BNP) was elevated at 501 (0–100 pg/cc). She was treated with intravenous furosemide and quickly improved.

**Figure 2:**
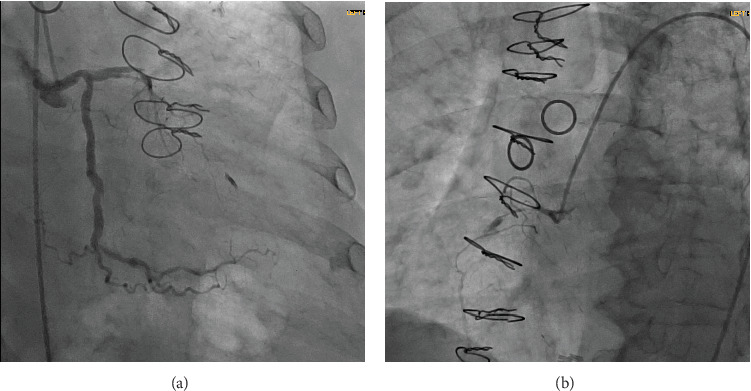
(a) Coronary angiogram showing severe native coronary artery disease of the left system. (b) Right coronary artery angiogram demonstrating a small codominant vessel with chronic total occlusion and left-to-right collaterals (from the left internal mammary artery injection) filling the small distal RCA vessel.

**Figure 3:**
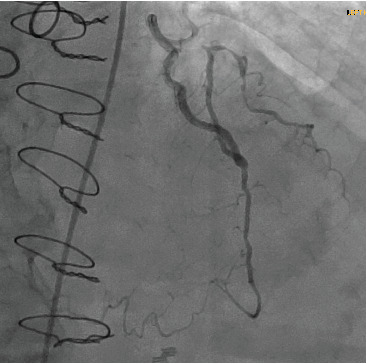
Left internal mammary artery (LIMA) angiogram showing LIMA to left anterior descending which was widely patent with brisk antegrade and retrograde flow filling a chronically diseased first diagonal branch and giving collaterals to distal right coronary artery.

The patient remained stable and was taken to the cardiac catheterization laboratory the following day. LAD showed proximal chronic total occlusion (CTO). Left circumflex was a codominant vessel with no obstructive disease. OM1 was chronically occluded, and OM2 had 50% ostial stenosis with TIMI-3 flow. RCA was a small codominant vessel with CTO and left-to-right collaterals (from the LIMA injection) filling the small distal RCA vessel (Figure 2a,b). LIMA to LAD was widely patent with brisk antegrade and retrograde flow filling a chronically diseased first diagonal branch and giving collaterals to distal RCA (Figure 3). SVG's to OM1 and RCA were occluded. Her coronary anatomy was felt to be stable with no identifiable acute or obstructive lesions to explain her event and the new WMA noted on her echocardiogram. She was treated medically and discharged home in a stable condition.

Upon follow-up, 2 weeks later, she reported doing well and a repeat TTE showed recovered LV function with normal LVEF and normal wall motion (Video S3).

## 3. Discussion

TC is an acute, usually transient, LV dysfunction with WMA typically involving apical and mid-ventricular segments, without acute, severe CAD to fully explain the presentation. Less commonly, the WMA may be limited to the midventricular walls or basal segments (reverse Takatsubo) [1, 3, 6, 7].

The incidence of TC has been steadily increasing, partly due to greater awareness of this condition. The disease may coexist with obstructive CAD in about 20%–25% of cases [1] and likely remains underdiagnosed in these patients.

TC has a well-established association with negative emotional stress. Mental health conditions, such as depression, panic, and anxiety, are present in 25%–40% of patients [2, 3, 6, 7]. In addition to emotional stress, TC is being increasingly recognized with physical stressors including heavy physical activity, acute illness, and certain medical or surgical procedures. [1, 3, 4, 6, 7] Such physical stressors are usually more prevalent among men [8] and are more likely to be associated with atypical forms of TC that affect the mid or basal LV segments [6]. Acute stressors and triggers, however, may not be readily identifiable in about 30% of patients, possibly due to different cultural backgrounds and coping skills by patients in addition to inadequate probing by providers [2–4, 6, 7].

TC typically mimics acute coronary syndrome (ACS) presentation with chest pain, acute shortness of breath, ECG changes and elevated biomarkers, and most patients may be misdiagnosed on presentation as having an ACS [8]. The ECG is usually abnormal, and changes may include ST elevation, T-wave inversion, QT prolongation and, less commonly, ST depression. [1, 3, 8, 9] The ST-T changes may not be localized to a specific distribution but are mostly seen in the precordial leads [9]. Troponin elevation is usually modest and disproportionately low relative to the degree of WMA and LV dysfunction [7].

Prognosis is usually good and better than ACS cases associated with similar degrees of LV dysfunction, with usually quick, spontaneous recovery of LV function in most cases [4, 5]. However, the disease is not benign with potential for cardiogenic shock and serious in-hospital complication rates comparable to ACS [3, 7]. Male gender, physical triggers, coexistent severe CAD, and the presence of dyspnea on presentation have all been associated with worse prognosis [1, 3, 6, 7].

We believe this may be the first well-documented case of TC secondary to a diagnostic MRI scan. The event occurred shortly after what the patient described as a fearful experience undergoing her first MRI scan ever. The temporal relationship to the stressful event-MRI was well documented with serial pre- and post-MRI echocardiograms, biomarkers, and EKGs. Our patient also had hyponatremia which was severe on presentation but only mild on the day of the event. Hyponatremia has been associated with TC, and although severe hyponatremia may act as a co-trigger, mild-moderate hyponatremia is more likely a facilitator or a bystander [10].

Although our patient had coexistent CAD, there were no new or acute culprit lesions to explain the WMA noted on echocardiography. In addition, her troponin elevation was disproportionately low relative to the degree of LV dysfunction and her LVEF quickly recovered on follow-up echocardiogram in 2 weeks.

This case highlights the importance of recognizing TC as a potential, albeit rare, complication of diagnostic MRI scans. While specific risk factors for TC in the context of noninvasive procedures like MRI remain unclear, this case underscores the need for clinical awareness, particularly in similar patients who may be more susceptible to stress-induced cardiomyopathy, such as postmenopausal women with heightened anxiety about the procedure, hyponatremia, and underlying psychiatric conditions.

## Data Availability

The data that support the findings of this study are available from the corresponding author upon reasonable request.

## Supporting Information

Supporting Information 1 Video S1 Initial transthoracic echocardiogram showing normal left ventricular ejection fraction and no wall motion abnormalities.

Supporting Information 2 Video S2 Transthoracic echocardiogram after MRI showing new finding of severe LV dysfunction with severe hypokinesis to akinesis of the apex and mid-distal anterior, septal, and lateral segments with sparing of basal walls.

Supporting Information 3 Video S3 Follow-up transthoracic echocardiogram 2 weeks later showing recovered LV function with normal LVEF and normal wall motion.
